# Intercenter validation of a knowledge based model for automated planning of volumetric modulated arc therapy for prostate cancer. The experience of the German RapidPlan Consortium

**DOI:** 10.1371/journal.pone.0178034

**Published:** 2017-05-22

**Authors:** Carolin Schubert, Oliver Waletzko, Christian Weiss, Dirk Voelzke, Sevda Toperim, Arnd Roeser, Silvia Puccini, Marc Piroth, Christian Mehrens, Jan-Dirk Kueter, Kirsten Hierholz, Karsten Gerull, Antonella Fogliata, Andreas Block, Luca Cozzi

**Affiliations:** 1 Dept. of Radiotherapy and Radiation Oncology Department, University Medical Center Hamburg-Eppendorf, Hamburg, Germany; 2 Radiation Oncology Department Dr. Rohn & Kollegen at Klinikum Dortmund, Dortmund, Germany; 3 Radiation Oncology Department, Klinikum Darmstadt GmbH, Darmstadt, Germany; 4 Radiation Oncology Group Practice, Strahlentherapie Bonn-Rhein-Sieg, Bonn, Germany; 5 Radiation Oncology Department, Helios Universitaetsklinikum, Witten/Herdecke University, Wuppertal, Germany; 6 Medical Physics Department, Klinikum Dortmund, Dortmund, Germany; 7 Radiation Oncology Department, University Medical Center Schleswig-Holstein, Campus Luebeck, Luebeck, Germany; 8 Radiotherapy and Radiosurgery Department, Humanitas Clinical and Research Center, Rozzano, Italy; North Shore Long Island Jewish Health System, UNITED STATES

## Abstract

**Purpose:**

To evaluate the performance of a model-based optimisation process for volumetric modulated arc therapy applied to prostate cancer in a multicentric cooperative group. The RapidPlan (RP) knowledge-based engine was tested for the planning of Volumetric modulated arc therapy with RapidArc on prostate cancer patients. The study was conducted in the frame of the German RapidPlan Consortium (GRC).

**Methods and materials:**

43 patients from one institute of the GRC were used to build and train a RP model. This was further shared with all members of the GRC plus an external site from a different country to increase the heterogeneity of the patient’s sampling. An in silico multicentric validation of the model was performed at planning level by comparing RP against reference plans optimized according to institutional procedures. A total of 60 patients from 7 institutes were used.

**Results:**

On average, the automated RP based plans resulted fully consistent with the manually optimised set with a modest tendency to improvement in the medium-to-high dose region. A per-site stratification allowed to identify different patterns of performance of the model with some organs at risk resulting better spared with the manual or with the automated approach but in all cases the RP data fulfilled the clinical acceptability requirements. Discrepancies in the performance were due to different contouring protocols or to different emphasis put in the optimization of the manual cases.

**Conclusions:**

The multicentric validation demonstrated that it was possible to satisfactorily optimize with the knowledge based model patients from all participating centres. In the presence of possibly significant differences in the contouring protocols, the automated plans, though acceptable and fulfilling the benchmark goals, might benefit from further fine tuning of the constraints. The study demonstrates that, at least for the case of prostate cancer patients, it is possibile to share models among different clinical institutes in a cooperative framework.

## Introduction

Knowledge based radiotherapy treatment planning (KBP in the following) is a concept pioneered since some years and the results published by the developers of the main algorithms [1–7] suggest the possibility to largely automate and individualize the definition of the appropriate dose-volume constraints for inverse planning. The basic idea is to develop clever engines which, after mining the historical planning data can predict the expected dose volume histograms (DVH) for any organ at risk (OAR) of any new patient. Different solutions have been proposed, from look-up engines, to machine learning based systems but all with the scope to automate one of the most critical and subjective phases of the inverse planning process. In fact, sub-optimal choice of the planning constraints might lead to sub-optimal final plans but the identification of the best (or best balanced) set of these might result a too hard task in the real world practice. Reasonably, limits in knowledge, expertise, resources and time constraints are the main risk factors for inadequate planning.

Among the solutions available for clinical practice, one commercial system, the RapidPlan (RP) has been already intensively investigated in literature [8–21]. In these studies some general trends were observed and included a generally improved plan quality, a reduced inter-clinician variability, and the possibility to transfer the planning expertise from more experienced centers to less experienced institutions. To mention that RP has been introduced in clinical practice and that Hussein et al [18] summarized well in their work the main features of the system and the main aspects of the construction of a predictive model. In summary, RP is an engine that takes the geometrical features of the patients and correlates these to the previously achieved dosimetry to generate appropriate estimates of the achievable dose distributions for prospective cases and the automated definition of the optimization constraints.

The German RapidPlan Consortium (GRC) was created as a cooperative initiative among six radiation oncology institutes through Germany with the scope to facilitate the learning phase of this new technology and to shorten the time needed to move from the pre-clinical to the clinical implementation of the KB process. Members of the GRC are both academic institutes and departments of regional community hospitals or of private networks of hospitals. The mix of different sizes, level of available infrastructures and human resources allowed to harmonize in the group the various types of clinical centres representative of the wider radiation oncology community. The activity of the GRC consisted, so far, in several tasks including: i) individual learning and assessment of the RapidPlan KBP system; ii) development, training and in-house validation (closed-loop) of some models per center; iii) cross validation of the models with other centres of the GRC (open-loop tests) and, more recently the execution of a multicentric experiment mimicking the possible broader sharing of a validated model.

Aim of this latter study, summarized in this report, was primarily to demonstrate the usability of a model, developed by one centre of the GRC by all other members with the addition of an external member with possibly different clinical characteristics.

The paradigmatic clinical case selected for the study was high risk prostate and the treatment with volumetric modulated arc therapy (VMAT) of the pelvic volume. Fogliata et al. [9] and Hussein et al [18] reported about the development of RP models for the prostate but for different settings from what presented here. Furthermore, the results presented in those studies are relative to single institute experience.

The experiment would have been considered positive if all the institutes would have been capable to generate plans acceptable with respect to the institutional goals and if the average over the entire cohort would fulfil the benchmark requirements with the RP system. A more quantitative assessment would also aim to determine the possible improvement in plan quality induced by the use of RP and/or determine the limits or weaknesses of the system.

## Material and methods

The RapidPlan model: a KB predictive model for RapidPlan was built for high risk prostate cancer patients, the model was designed for the treatment of the pelvic volume (including the prostate, the seminal vesicles and the pelvic nodes, partial treatment and no simultaneous boost concept applied). The dose prescription was set to 50.4Gy (in 28 fractions). All plans were normalised at the mean target dose. The OARs considered for the model definition and training were the bladder, the rectum, the femoral heads and the small intestine. The contouring of the small intestine actually accounted for the bowel bag, trying to encompass all possible positions of the intestinal loops. The margins from the clinical treatment volume (CTV) to the planning treatment volume (PTV) was defined as 7mm isotropic.

The model was trained with a set of 43 patients all selected by a single institute of the Consortium using a TrueBeam linear accelerator (Varian Medical Systems, Palo Alto, US) with the VMAT RapidArc technique and 2 full arcs. The photon beam energy selected for the model training dataset was 15MV but this did not constitute in the study design a mandatory requirement for the tests. The dose plan optimisation, calculation and the model training were performed using the corresponding algorithms of the Eclipse planning system version 13.6.23. The model validation was performed by inspection of the potential outliers and the quality of the treatment plans selected for the training. The final model version, approved for the study, resulted exempt the presence of any major outlier potentially influencing (negatively) the estimation power of the model.

The objectives defined for the DVH constraints definition are summarized in Table 1.

**Table 1:** constraints and objectives as defined in the RapidPlan model.

Type	Volume (%)	Dose (%)	Priority
**CTV**			
Upper	0	100	100
Lower	100	99	100
**PTV**			
Upper	0	105	150
Upper	0	100	100
Lower	100	98	150
Lower	100	100	120
**Bladder**			
Upper	0	100	Generated
Line	Generated	Generated	Generated
**Rectum**			
Upper	0	100	Generated
Line	Generated	Generated	Generated
**Small Bowel**			
Upper	0	50 (Gy)	Generated
Line	Generated	Generated	Generated
**Femoral Heads (left or right)**			
Upper	0	40 (Gy)	Generated

The inter-center test: the model was distributed among all the Consortium centers plus one additional site out of the core group and applied to a number of patients per site, these centers are labelled S1 to S7 and include also the center where the model was built. A total of 60 test plans were developed based on this model (the number of cases per center were 10,7,6,7,13,10,7 respectively).

Each RapidPlan case was compared against a corresponding plan, manually optimized.

To provide a “real world” assessment of the robustness of the DVH prediction power of the RP model, each center was requested to include in the study typical patients belonging to the high risk category but without any modification of the local contouring strategies. While the technique (2 full RapidArcs) was requested to be kept for all cases, the beam energy was left free and it was selected to be either 6MV or 15MV according to local practice.

The RapidPlan optimization was performed for all 60 test cases without any interactive intervention on the process. No additional control structures were used for the optimization.

The manually optimized plans, were instead designed according to the standard procedures of the individual clinics, with the possible use of help structures and with interactive modification of the constraints if needed. Although the clinically acceptable dose-volume constraints would depend on the single individual center, the general benchmark objectives to be met for the average of the entire cohort were defined as follows. For the target coverage D_98%_≥98% (95%) for CTV (PTV respectively). For the organs at risk, the maximum dose D_1%_ to the bowel and the femoral heads was required to be inferior to 50 (40)Gy respectively. For the rectum the constraints were mean dose <36Gy, V_50%_<10% and for the bladder mean dose <36Gy and V_50Gy_<20%; D_1%_<50Gy was the ideal objective, not possible to meet due to the overlap between PTV and bladder or rectum, so an alara principle was applied in this case.

Data analysis: all DVHs from all 60 test cases were exported from Eclipse and centrally analysed by one of the sites using the same metrics and calculation tools for all. Standard quantitative and qualitative assessment of the DVH was performed by inspecting a number of dose-volume parameters for either the targets (aiming to coverage and homogeneity information) or for the OARs (aiming to meaningful metrics for organs sparing). In particular, the Conformity Index (CI) was defined as the ratio between the body volume covered by the 95% isodose and the volume of the PTV while the Homogeneity was measured as the difference between D_5%_ and D_95%_ divided by the meann dose to the PTV.

Boxplots used to report differences between the various datasets reports five statistics: the median (solid line), the first and third quartile (bottom and top ends of the box) and the whiskers which correspond to 1.5 times the height of the box or, if not cases fall in that range, the minimum or maximum values (for normally distributed data this should correspond to approximately the 95% confidence interval) for each parameters. The points possible represented outside the limits of the whiskers are the outliers.

## Results

**Fig 1:**
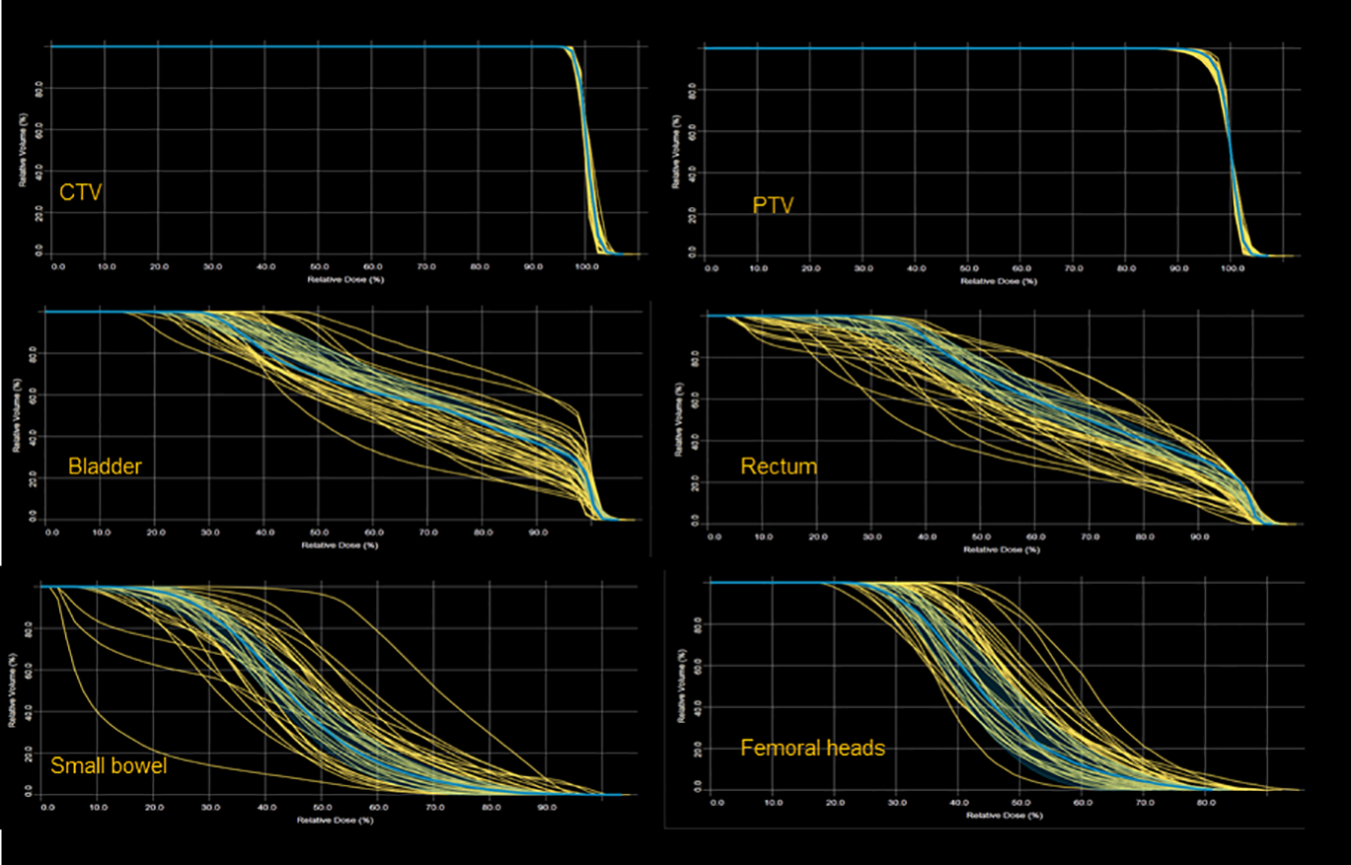
Distribution of the dose volume histograms of each structure used for the model definition from the 43 patients used for the training. Outlined one typical example with the bands showing the prediction variance inherited from the training cohort.

To appraise the quality of the input cases used for the model construction and training, Fig 1 presents the distribution of DVHs for the various structures (targets and OARs) used for the model training with a typical case outlined together with its prediction band. The most spread-out group was the small bowel as reasonably expected given the contouring broad definition.

**Fig 2:**
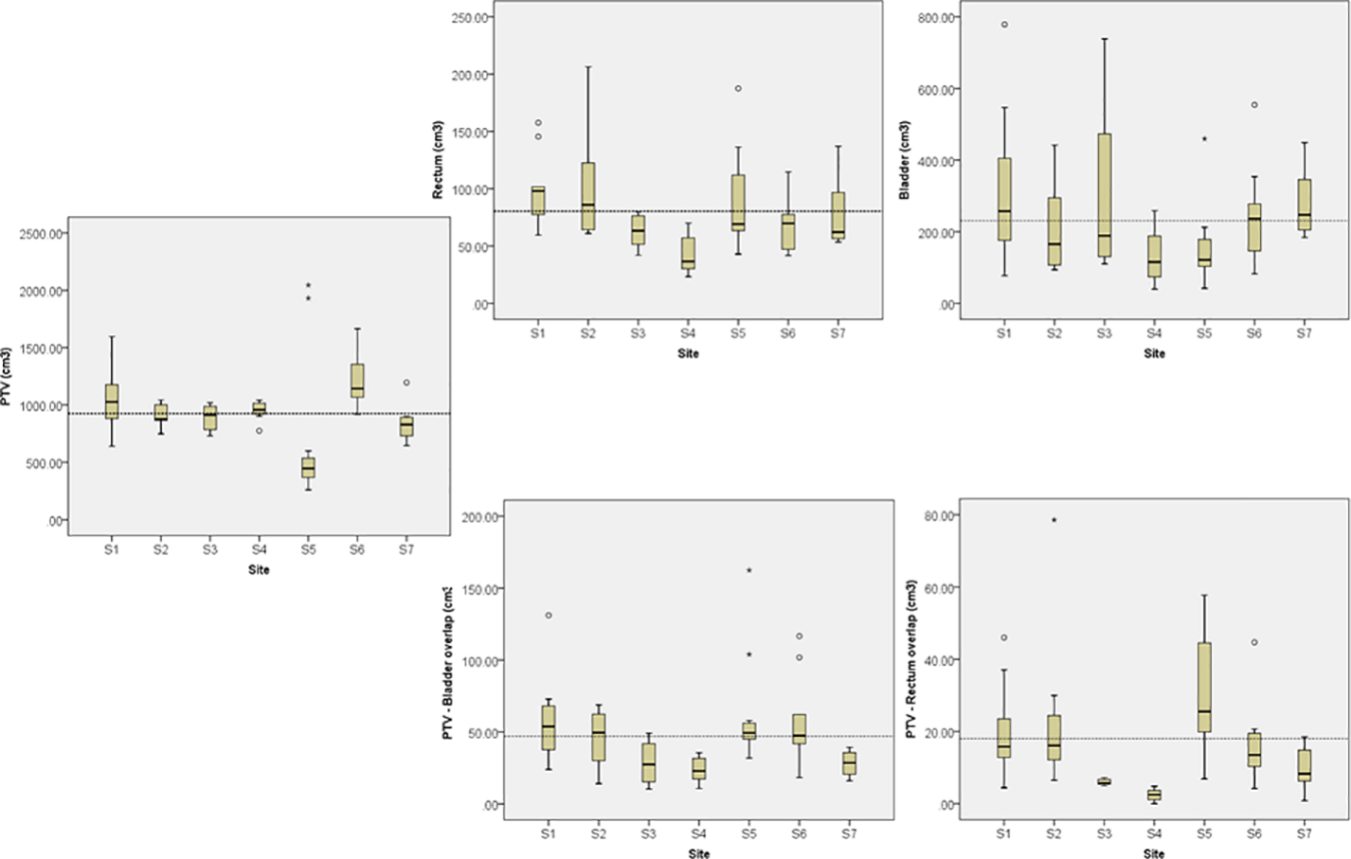
Summary of the analysis of the volume differences for PTV, bladder and rectum among the seven centres participating to the study. The dashed line represents the average value over the cohort. PTV and rectum distributions resulted significantly different among the groups while this did not occurred for the bladder.

The heterogeneity of the test population was appraised in terms of the variability of the volumes of the targets and the two main organs at risk (bladder and rectum). Fig 2 shows the boxplots from the analysis of the volumes of the PTV, the bladder and the rectum. The dashed line represents the median value for the entire cohort of 60 patients. As it can be noted, there have been significant differences in the contouring strategy for the PTV as well as for the rectum, both statistically significant with a one-way analysis of variance (p = 0.01 and p = 0.02 respectively) while the observed difference for the bladder were not statistically significant (p = 0.09). The overlap volume between PTV and bladder or organ resulted significantly different (p<0.001 for rectum and p = 0.01 for bladder) when analysed in absolute or in percentage terms.

**Fig 3:**
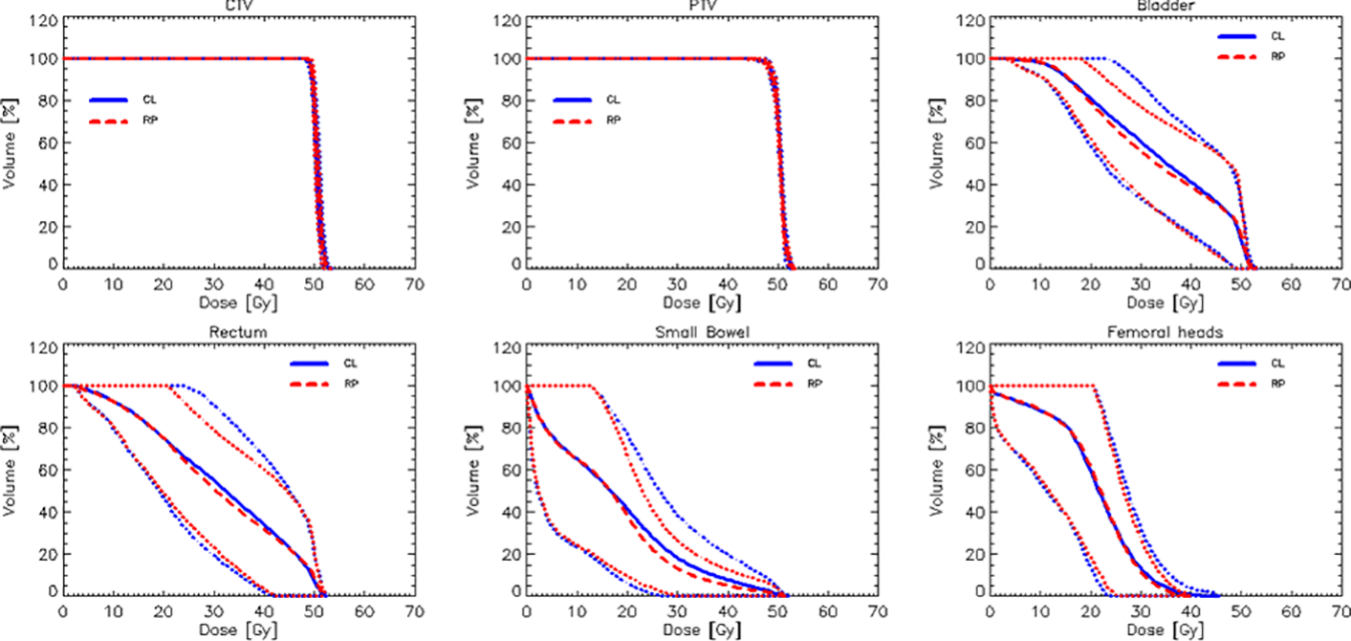
Average dose volume histograms for the entire cohort of 60 patients comparing RapidPlan based (RP) vs. manually optimized (CL) plans. The solid (dashed) lines are the averages for the CL (RP) data while the inter-patient variability at 1.5 standard deviation is represented by the dotted lines. On average the RP plans proved to be moderately better than the CL plans in the mean to high dose range.

**Fig 4:**
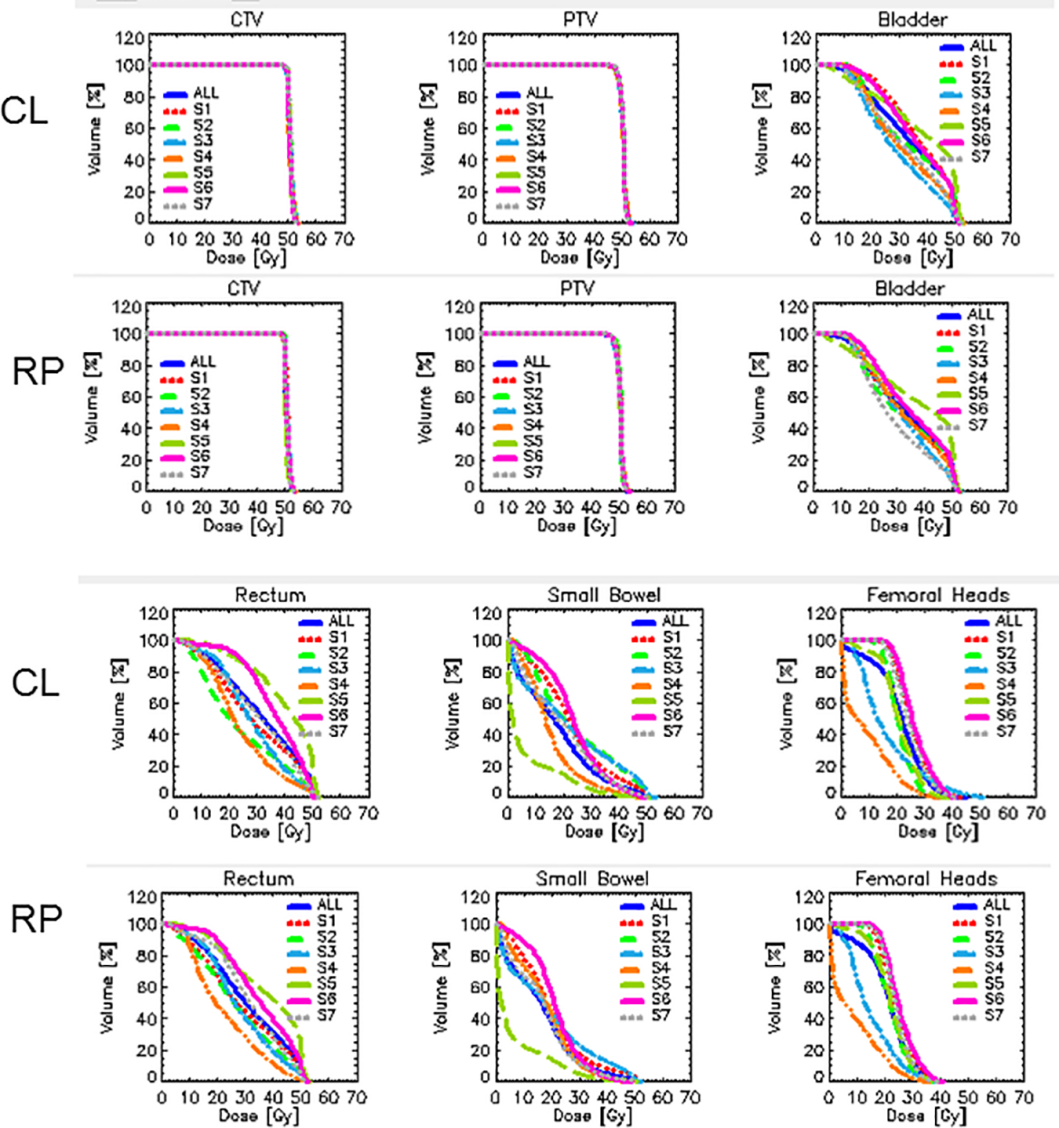
Dose volume histograms per target volume and organ at risk averaged per each participating centre. The graphs shows the significant variation in the level of sparing of all OARs corresponding to different contouring and optimization strategies. The variance is substantially consistent between RP and CL plans.

Fig 3 shows the average DVH for the whole cohort of patients comparing the RP based and the manual (CL) plans. The dashed lines represent the 1.5 standard deviation variance. Fig 4 shows the average DVHs per structure and per center separated for RP and CL plans and illustrates the wide range of OAR sparing (consistent with the range of volumes and overlapping fractions) while the coverage and homogeneity of the dose to the CTV and PTV resulted highly consistent between all centers. In the figure also the graphs for the entire cohort are represented (and labelled as All). The numerical analysis of the DVH is summarized in Table 2 for the CTV and the PTV and in Table 3 for the various OARs. CI was of course defined and reported only for the PTV. The data are reported for each site individually as well as for the entire cohort (All) and expressed as mean values while the interpatient variability is reported at 1 standard deviation level.

**Table 2:** Summary of the DVH analysis for the CTV and the PTV.

**CTV**										
Center	Mean		D_2%_		D_98%_		Homogeneity		CI	
	CL	RP	CL	RP	CL	RP	CL	RP	CL	RP
S1	50.6±0.2	50.8±0.1*	52.3±0.4	52.1±0.3	48.7±0.4	49.5±0.2	0.05±0.01	0.04±0.01	-	-
S2	50.6±0.1	50.3±0.1*	51.9±0.2	51.7±0.1*	49.3±0.2	49.4±0.1	0.04±0.01	0.04±0.01	-	-
S3	50.7±0.1	50.7±0.1	52.7±0.3	52.4±0.1	48.9±0.2	49.3±0.1*	0.06±0.01	0.05±0.01	-	-
S4	50.6±0.1	50.6±0.1	52.8±0.2	52.6±0.2	49.0±0.1	49.0±0.1	0.06±0.01	0.06±0.01	-	-
S5	50.6±0.3	50.2±0.2*	51.9±0.6	51.6±0.5*	49.3±0.4	49.2±0.2	0.04±0.01	0.04±0.01	-	-
S6	50.6±0.1	50.6±0.1	52.1±0.2	52.4±0.2	49.2±0.3	49.2±0.1	0.05±0.01	0.05±0.01	-	-
S7	50.8±0.1	50.5±0.2*	51.8±0.5	52.0±0.3	49.8±0.3	49.4±0.2*	0.03±0.01	0.04±0.01	-	-
All	50.6±0.2	50.5±0.2*	52.2±0.5	52.1±0.5	49.2±0.4	49.3±0.2	0.05±0.01	0.04±0.01	-	-
**PTV**										
Center	Mean		D_2%_		D_98%_		Homogeneity		CI	
	CL	RP	CL	RP	CL	RP	CL	RP	CL	RP
S1	50.4±0.0	50.4±0.0	52.4±0.3	52.2±0.2*	48.1±0.5	47.4±0.5*	0.07±0.01	0.07±0.01	1.04±0.01	1.04±0.01
S2	50.4±0.0	50.4±0.0	51.9±0.1	52.1±0.1*	47.9±0.2	48.3±0.2*	0.06±0.01	0.05±0.01	1.03±0.01	1.03±0.01
S3	50.4±0.0	50.4±0.0	52.7±0.2	52.6±0.1	47.7±0.4	47.3±0.1	0.07±0.01	0.08±0.01	1.04±0.01	1.04±0.01
S4	50.4±0.0	50.4±0.0	52.9±0.1	52.8±0.1	47.3±0.3	47.4±0.2	0.08±0.01	0.08±0.01	1.03±0.01	1.03±0.01
S5	50.4±0.0	50.4±0.0	52.1±0.4	52.5±0.2*	47.2±0.9	47.7±0.7	0.07±0.01	0.07±0.01	1.02±0.01	1.02±0.01
S6	50.4±0.0	50.4±0.0	52.4±0.2	52.9±0.2*	47.7±0.3	47.5±0.1	0.07±0.01	0.08±0.01	1.06±0.01	1.06±0.01
S7	50.4±0.0	50.4±0.0	51.9±0.5	52.3±0.2*	48.0±0.5	47.8±0.2	0.06±0.01	0.07±0.01	1.03±0.01	1.03±0.01
All	50.4±0.1	50.4±0.1	52.3±0.5	52.5±0.3	47.6±0.6	47.6±0.5	0.07±0.01	0.07±0.01	1.03±0.02	1.03±0.02

Sx: center number, CL: clinical plan; RP: RapidPlan based plan. D_2%_: dose received by at least 2% of the volume, D_98%_ dose received by at least 98% of the volume; CI: conformity index.

* = statistically significant difference (p< = 0.05)

**Table 3:** Summary of the DVH analysis for the various OARs.

	**Bladder**									
	**Mean**		**D1%**		**V40Gy**		**V45Gy**		**V50Gy**	
	**RP**	**CL**	**RP**	**CL**	**RP**	**CL**	**RP**	**CL**	**RP**	**CL**
S1	37.1±4.8	33.1±4.3*	51.9±0.6	51.5±0.4*	36.3±13.8	45.3±14.2*	28.4±13.8	33.7±13.8*	0.0	0.0
S2	33.1±3.5	32.1±3.7*	51.6±0.3	51.8±0.3	36.0±10.7	39.5±11.4*	29.1±10.4	31.7±11.2*	13.7±6.4	14.9±7.0*
S3	29.0±4.2	31.8±4.3*	51.1±0.6	51.7±0.9*	29.1±14.2	25.0±13.7*	18.9±13.7	16.3±12.2	7.3±8.3	3.4±1.7*
S4	31.7±2.7	33.1±2.5	52.4±0.5	52.1±0.4	35.3±10.0	30.8±8.8*	26.4±10.5	22.7±8.3*	13.5±8.7	10.5±4.5
S5	37.3±7.0	36.7±6.5	51.8±.04	51.9±0.4	54.2±17.2	54.9±19.7	48.2±16.7	47.8±17.6	28.3±10.5	28.6±13.0
S6	36.1±4.0	35.3±3.4	51.0±0.2	51.6±0.3*	41.6±12.6	43.4±12.6	32.6±13.1	32.7±12.4	14.6±8.4	7.3±3.5*
S7	29.4±3.1	31.4±3.5	51.4±0.5	51.4±1.1*	25.0±7.7	34.1±10.2*	17.6±6.6	23.4±8.4*	6.3±3.6	5.9±4.0
All	34.2±5.7	33.6±4.8*	51.6±0.7	51.7±0.5	38.9±15.8	41.4±16.4*	31.0±16.1	32.1±16.0	15.7±11.0	13.8±11.7*
	**Rectum**									
	**Mean**		**D1%**		**V40Gy**		**V45Gy**		**V50Gy**	
	RP	Man	RP	Man	RP	Man	RP	Man	RP	Man
S1	29.3±4.7	28.5±4.9*	51.8±0.8	51.4±0.9*	42.3±5.9	43.5±5.3	28.0±13.9	30.3±15.0*	7.6±5.4	8.9±5.8
S2	23.7±4.0	26.8±3.7*	50.7±1.3	51.0±0.7	40.4±4.5	38.8±5.7	22.1±8.7	20.0±9.1	4.3±2.8	3.4±2.0*
S3	28.2±1.6	27.9±0.1	50.5±0.8	51.4±0.3	39.5±1.4	38.9±1.3	19.2±2.2	18.1±2.2	4.1±1.0	2.0±0.9*
S4	24.0±5.0	22.4±4.8	49.0±4.7	48.6±4.9	31.8±8.6	33.7±7.9	11.4±7.0	12.5±8.8	1.4±1.5	2.5±2.3
S5	37.9±5.6	37.2±5.4*	51.6±0.5	51.9±0.6	49.4±1.4	49.2±1.4	52.4±19.2	59.1±20.4	20.8±8.4	19.6±12.3
S6	36.3±4.5	35.1±4.5	50.1±0.5	51.4±0.5*	46.0±3.9	45.4±3.0	39.9±17.1	42.6±19.4	9.4±6.5	1.6±1.2*
S7	31.5±3.3	30.2±3.5	50.6±1.7	50.3±1.7*	43.2±3.3	43.2±3.6	28.5±9.5	31.3±10.3	4.3±2.0	3.4±3.0
All	31.6±7.2	30.7±6.4*	50.7±1.9	51.1±1.9*	42.9±6.8	42.9±6.4	31.8±18.8	34.2±21.4*	8.9±8.5	7.3±9.3*
	**Femoral heads**				**Bowels**					
	**Mean**		**D1%**		**Mean**		**D1%**		**V50Gy**	
	**RP**	**Man**	**RP**	**Man**	**RP**	**Man**	**RP**	**Man**	**RP**	**Man**
S1	25.1±2.8	24.3±2.5	37.6±4.6	37.5±2.1	23.2±5.1	20.1±4.1*	47.6±6.9	47.2±7.3	0.0±0.0	0.0±0.0
S2	20.6±2.5	21.6±2.8	30.8±3.4	33.2±2.4	15.1±2.5	14.9±3.2	46.0±4.9	45.6±5.8	0.0±0.0	0.0±0.0
S3	16.6±2.7	14.8±1.3	45.4±4.1	36.2±1.0*	21.8±5.8	19.0±4.9*	51.4±1.1	50.9±0.4	0.0±0.0	0.0±0.0
S4	8.9±0.8	8.9±1.1	31.4±2.5	31.9±2.7	15.4±1.7	17.9±1.8*	43.3±2.9	41.9±3.5	0.0±0.0	0.0±0.0
S5	20.6±5.7	22.0±4.9	29.6±5.7	32.8±4.5*	6.9±7.6	7.0±8.1	34.5±14.6	30.8±15.4*	0.0±0.0	0.0±0.0
S6	26.3±1.6	25.4±1.6	39.1±0.9	38.6±1.2	23.9±3.5	21.4±2.3*	46.1.±2.7	43.1±3.4*	0.0±0.0	0.0±0.0
S7	25.1±1.4	23.3±2.1	37.5±2.6	34.8±2.7*	16.8±4.4	19.1±4.6*	46.6±2.4	47.7±2.2*	0.0±0.0	0.0±0.0
All	21.1±6.2	20.8±5.9	35.4±6.3	35.1±3.6	17.7±7.7	16.6±6.6*	44.6±8.8	43.1±9.6*	0.0±0.0	0.0±0.0

Sx: center number, CL: clinical plan; RP: RapidPlan based plan. D_1%_: dose received by at least 1% of the volume, V_xGy_ volume receiving at least x Gy.

* = statistically significant difference (p< = 0.05)

Concerning the target volumes (Table 3), no remarkable difference was observed among the parameters in terms of homogeneity and conformity. Some differences were reported to be statistically significant (p<0.05, marked with a *). Nevertheless, the absolute difference observed in those cases is small. For example we reported a difference of 0.2–0.4Gy (i.e. ~0.5–1% of the prescribed dose) for the mean dose to the CTV or 0.4Gy for (0.8%) for D98%. In general all RP or CL plans resulted equivalent.

**Fig 5:**
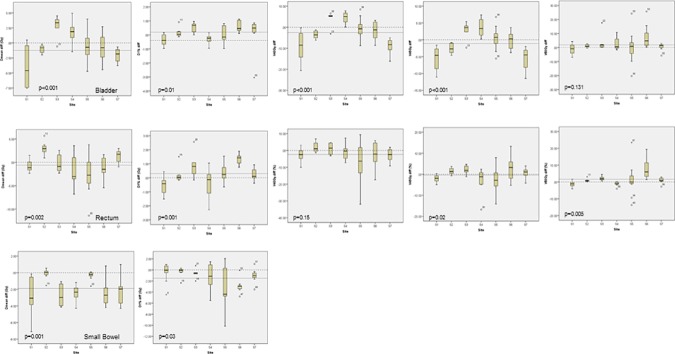
The absolute difference (in Gy or in %) between RP and CL plans for the bladder (a), the rectum (b) and the small bowel (c) for a subset of significant metrics. Negative values in the boxplot represents an improvement derived from the use of RP. The dotted line represents the median over the entire cohort while the dashed line at 0.0 difference is the neutral case.

The analysis of the OARs data is less straightforward and reflects the different clinical strategies and preferences among the various centers (in the CL sets) as well as the impact of the different contouring rules (and therefore different overlaps). This can be better visualized by Fig 5 where the boxplots of some relevant metrics are shown for the OARs. The data shown are the absolute differences (in Gy or in % of the volumes) between the RP and the CL plans. A negative value means an improvement achieved with the RP plan. Fig 5A reports the findings for the bladder, Fig 5B for the rectum and Fig 5C for the small bowel. In each plot the dashed line at 0.0 difference represents the neutral case while the other dashed line reports the median difference for the entire cohort. The p values reported in the figure are the significance of the differences observed after the one-way analysis of variance.

The mean dose to the bladder improved, on average, of 0.8Gy; the improvement was of 2.5% for V_40Gy_ and of 1.1% for V_45Gy_; a 0.9Gy and 1.9% worsening were observed for D_1%_ and V_50Gy_. For the rectum an average improvement of 0.6Gy was for the mean dose and of 2.4 and 0.4% for V_40Gy_ and V_45Gy_ while D_1%_ and V_50Gy_ worsened of 0.3Gy and 1.6% respectively. Lastly, for the small bowel, the average improvement for the mean dose was of 1.9Gy while it resulted of 1.5Gy for D_1%_. Despite the significant differences in the degree of overlap between PTV and bladder or rectum observed among centers, no further correlation was found between the degree of overlap and any dosimetric parameter.

**Fig 6:**
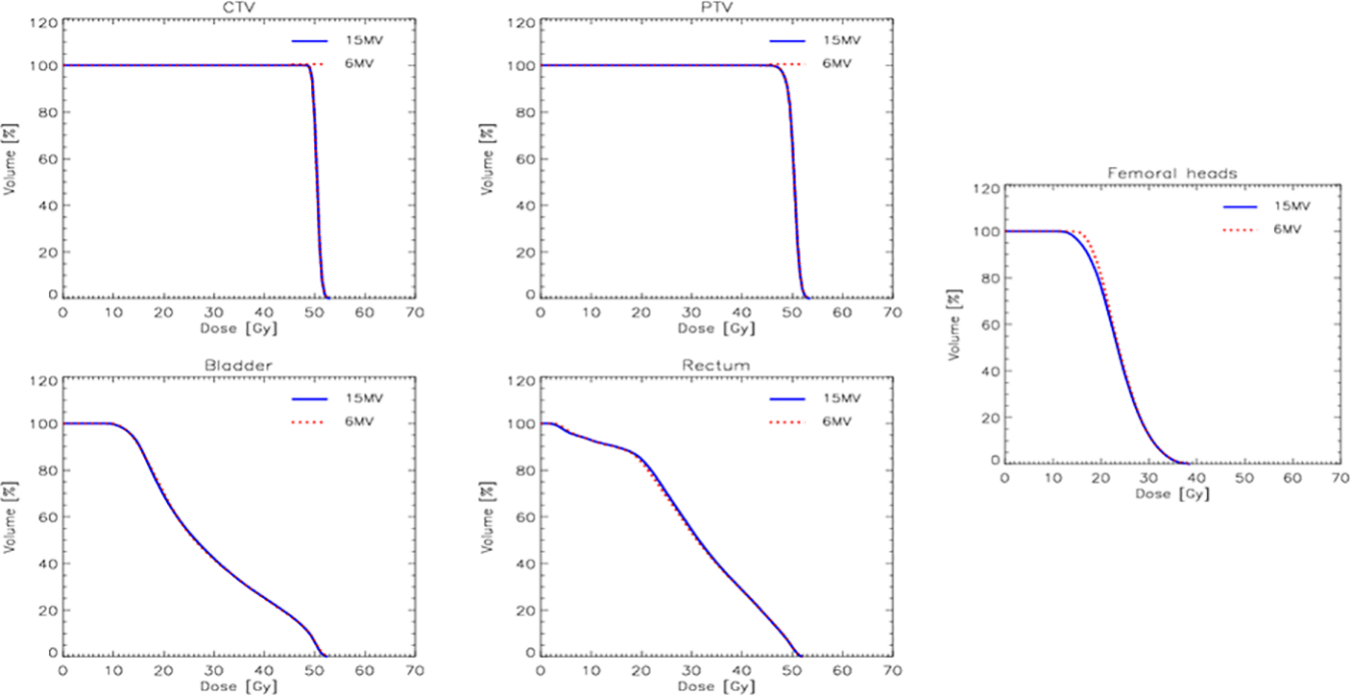
The comparison of the average dose volume histograms for the target volumes and the organs at risk between RP plans optimised for 6 or 15 MV photon beams.

A side cross-validation test was performed to validate the usability of the same model irrespective of the beam energy selected for the plans. The average DVH for the target volumes and some of the organs at risk from one center are shown in Fig 6. No differences were observed between the plans optimised for 6 or 15 MV photons and all based on the same model.

## Discussion

Scope of the investigation was to understand if a knowledge based model aiming to automate inverse planning procedures, developed in one center was effectively applicable to other institutions of the same or near-to-same geographical and cultural area. The “affinity” among the centres would imply reasonable similar practice, protocols and some homogeneity in the patients´ population. The model analysed aimed to be a broad scope one for the treatment of the pelvic volume in the high risk group of prostate cancer patients. No special conditions were imposed to the testing centers to strictly adhere to the model definitions (in terms of contouring rules for example) but rather the scope of the study was to appraise the possibility to use the same model within a ‘real world’ environment mimicking daily practice in different types of institutes. The institutes belonging to the GRC ranged from relatively small private departments (members of a network) to public regional community hospitals to finally larger academic centers. The results demonstrated that, on average, the use of KBP tools allowed some improvement in the sparing of the organs at risk compared to the routine clinical practice without compromising the coverage of the target volumes. At a site-per-site analysis, the performance of the automated planning revealed some different flavours and this has been reported primarily to two factors: the heterogeneity in the contouring rules (i.e. an heterogeneity in the “patients” set) and different logical priorities in the sparing of the OARs (e.g. some centres emphasising bladder over rectum or vice versa).

A significant metric to prove the heterogeneity of the test dataset was provided by the variability of the overlap between the PTV and the bladder or the rectum. This from one side makes evident the different contouring protocols even within culturally homogeneous groups and on the other side justifies to some extent the differences observed in the degrees of sparing of the whole OARs as reported in the results. Indeed, the RapidPlan system used for the study, does not fully account for the eventual overlapping region giving priority to the target volumes in the generation of the objective lines and constraints.

The findings reported here confirm the quite obvious need of an accurate review and validation when a KB model generated and tested by other clinics is going to be applied for the first time in any new institute. Any un-supervised application of a KB model (or in general a technique not developed in-house) should be discouraged.

Berry et al [20] published an interesting study aiming to investigate whether the use of KBP would have allowed to identify systematic variation in IMRT planning between different satellites of the same institute. The study was performed for intensity modulation treatment of esophageal cancer and a model built at the main campus was distributed to the others for testing and benchmarking. Their results proved that this was the case and that the use of KBP allowed to identify differences among the campuses possibly due to different levels of expertise, workload or preferences. Both Berry’s and ours findings are consistent and from both it can be derived that KBP methods can be used either to harmonize practice or to facilitate the identification of challenges. In any case, KBP can lead to improvements in the planning quality.

Li et al [21] aimed to investigate whether the use of KBP could facilitate the adherence to clinical trial requirements and also improve the quality of the plans based on the assumption that lack of quality control is one relevant factor impacting on the outcome of the trials. This is in line to a recent publication where it was shown that the clinical outcome is strongly correlated with the volume of patients treated in an institute [22]. Li demonstrated that, properly built, the use of a model allowed to outperform manual planning in all protocol-specific dose volume objectives. In the frame of our study, this implies that, in a multicentric cooperative initiative, the adherence to guidelines or to recommendations could be facilitated and made stronger by the use of KBP methods.

One unconventional feature of the present study is the mix of cases planned for 6MV or for 15MV photons. Despite the apparent inconsistency, this was felt to be a strength because primarily it proves the possibility to use the same model applied to somehow different strategies, even at the level of energy and still be adequate in the quality of the results. More specifically, the energy selection should not constitute a problem with VMAT plans where full arcs are adopted as in the present study since the quality of the plans results very much comparable as it was demonstrated quite early in the RapidArc era [23–24]. To further demonstrate the robustness of the model with respect to the energy selection, one centre generated two sets of plans with RP for 6 or 15MV photon beams and the results, not shown in this report for contingent reasons, demonstrated the complete equivalence of the two sets as illustrated in Fig 6.

## Conclusions

The multicentric validation demonstrated that it was possible to satisfactorily optimize patients from all participant centers with the knowledge based model. In the presence of possibly significant differences in the contouring protocols, the automated plans, In the presence of possibly significant differences in the contouring protocols, the automated plans, though acceptable and fulfilling the benchmark goals, might benefit from further fine tuning of the constraints.
